# Statistical investigation of the full concentration range of fasted and fed simulated intestinal fluid on the equilibrium solubility of oral drugs

**DOI:** 10.1016/j.ejps.2017.10.007

**Published:** 2018-01-01

**Authors:** Jeremy Perrier, Zhou Zhou, Claire Dunn, Ibrahim Khadra, Clive G. Wilson, Gavin Halbert

**Affiliations:** Strathclyde Institute of Pharmacy and Biomedical Sciences, University of Strathclyde, 161 Cathedral Street, Glasgow G4 0RE, United Kingdom

**Keywords:** BCS, biopharmaceutics classification system, DoE, design of experiment, FASSIF, fasted simulated intestinal fluid, FESSIF, fed simulated intestinal fluid, IVIVC, in vitro in vivo correlation, GIT, gastrointestinal tract, API, active pharmaceutical ingredient, Felodipine (Pubchem CID: 3333), Fenofibrate (Pubchem CID: 3339), Probucol (Pubchem CID: 4912), Indomethacin (Pubchem CID: 3715), Phenytoin (Pubchem CID: 1775), Aprepitant (Pubchem CID: 6918365), Tadalafil (Pubchem CID: 110635), Carvedilol (Pubchem CID: 2585), Zafirlukast (Pubchem CID: 5717), Equiibrium solubility, Fassif, Fessif, Design of experiment, Biopharmaceutics classification system

## Abstract

Upon oral administration the solubility of a drug in intestinal fluid is a key property influencing bioavailability. It is also recognised that simple aqueous solubility does not reflect intestinal solubility and to optimise in vitro investigations simulated intestinal media systems have been developed. Simulated intestinal media which can mimic either the fasted or fed state consists of multiple components each of which either singly or in combination may influence drug solubility, a property that can be investigated by a statistical design of experiment technique. In this study a design of experiment covering the full range from the lower limit of fasted to the upper limit of fed parameters and using a small number of experiments has been performed. The measured equilibrium solubility values are comparable with literature values for simulated fasted and fed intestinal fluids as well as human fasted and fed intestinal fluids. The equilibrium solubility data range is statistically equivalent to a combination of published fasted and fed design of experiment data in six (indomethacin, phenytoin, zafirlukast, carvedilol, fenofibrate and probucol) drugs with three (aprepitant, tadalafil and felodipine) drugs not equivalent. In addition the measured equilibrium solubility data sets were not normally distributed. Further studies will be required to determine the reasons for these results however it implies that a single solubility measurement without knowledge of the solubility distribution will be of limited value. The statistically significant media factors which promote equilibrium solubility (pH, sodium oleate and bile salt) were in agreement with published results but the number of determined significant factors and factor interactions was fewer in this study, lecithin for example did not influence solubility. This may be due to the reduction in statistical sensitivity from the lower number of experimental data points or the fact that using the full range will examine media parameters ratios that are not biorelevant. Overall the approach will provide an estimate of the solubility range and the most important media factors but will not be equivalent to larger scale focussed studies. Further investigations will be required to determine why some drugs do not produce equivalent DoE solubility distributions, for example combined fasted and fed DoE, but this simply may be due to the complexity and individuality of the interactions between a drug and the media components.

## Graphical abstract

**Figure:**
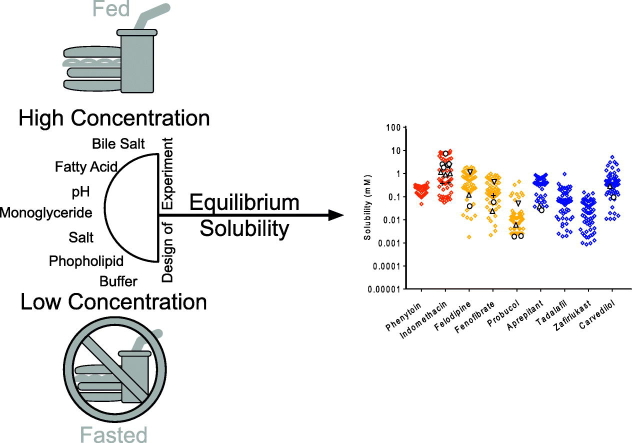

## 1 Introduction

Dissolution and solubility are essential parameters in the absorption process of orally administered drugs and especially for poorly soluble drugs (BCS class II and IV). Over the last two decades there has been an increasing development of molecules with low aqueous solubility due to the application during development of high throughput screening systems (Lipinski et al., 2001). Therefore, it is necessary to develop new formulation techniques in order to address this issue (Savjani et al., 2012) along with in vitro methods to predict drug solubility in gastrointestinal fluids (Lennernas et al., 2014). High throughput solubility screening is possible (Alsenz and Kansy, 2007) but a low aqueous solubility does not automatically mean poor gastrointestinal solubility. The solubilizing potential of the gastrointestinal environment can improve the bioavailability for some drugs over that predicted on the basis of simple aqueous solubility (Sunesen et al., 2005). For example, it has been reported that mixtures of bile salts increase the solubility of steroid formulations (Mithani et al., 1996, Wiedmann et al., 2002) and the interaction of lecithin with bile salts yields an even greater positive solubility effect (Naylor et al., 1993). Solubilisation can be further influenced by the formation of mixed micelles with other lipid digestion products such as monoglycerides and the interaction of monoglycerides with bile salt was demonstrated to increase the solubility of alpha-tocopherol in comparison to bile salt alone (Nielsen et al., 2001). To address the problem of poor aqueous solubility and bioavailability for oral drug formulations, it is therefore essential to use solubility and dissolution test conditions which closely reproduce key parameters of human gastrointestinal physiology (Dressman et al., 2007).

Over the past two decades simulated gastrointestinal media for the human fasted and fed states have been developed to assist in vitro drug development and formulation studies (Markopoulos et al., 2015, Stappaerts et al., 2014). These media were based around available literature data on the detailed composition and physicochemical parameters of human GI fluid however, the gastrointestinal tract and the interactions of all its constituents is very complex. To assess these interactions and improve the determination of the pivotal factors influencing the intestinal solubility of BCS class II drugs, a statistical design of experiment (DoE) approach was applied to investigate the influence of simulated gastrointestinal media composition in the fasted (Khadra et al., 2015) and fed state (Zhou et al., 2017) on the equilibrium solubility of BCS II compounds. This illustrated the utility of this approach, provided solubility values that are in agreement with literature values and highlighted the differences in solubility between the fasted and fed state (Augustijns et al., 2014, Bevernage et al., 2010, Clarysse et al., 2011). In addition, the approach simulated the inherent solubility variability and determined the key parameters controlling a drug's solubility. For acidic compounds pH was the most significant factor. For basic and neutral drugs the combination of pH and concentration of sodium oleate, bile salt and lecithin was significant. Various interactions between media components and unusual drug specific solubility behaviour were also identified. For neutral drugs solubilisation in fed simulated media was a more complicated interplay since seven (pH, oleate, bile salt, lecithin, monoglyceride, buffer and pancreatin) out of the eight single factors were significant along with more than half of the factor interactions.

In this paper the design of experiment approach has been applied to explore the equilibrium solubility of BCS class II drugs in simulated media spanning the full range of both fasted and fed intestinal states in a single experiment. The purpose is to examine the feasibility of merging the individual fasted and fed studies into one reduced experiment in order to obtain comparable results from a smaller experimental load. In this full range DoE the simulated intestinal fluid consists of seven factors or parameters (sodium oleate, bile salt, pH, lecithin, buffer, salt and monoglyceride) with phosphate buffer used instead of maleic acid. A fractional factorial design with two levels (upper and lower limit) was applied requiring a total of thirty two measurements and conducted in duplicate. This gives a total of 64 measurements for the statistical analysis. The lower limit values are derived from the lower limits of the literature fasted study (Khadra et al., 2015) and the upper limits are from the upper limits of fed study (Zhou et al., 2017) (Table 1). A smaller scaled DoE was selected in order to assess the utility of this systematic approach with a limited number of measurements. The equilibrium solubility of nine BCS class II drugs was investigated, two acids (indomethacin and phenytoin), four bases (aprepitant,^1^ tadalafil, zafirlukast and carvedilol) and three neutral drugs (felodipine, fenofibrate, probucol) and compared to the previous fasted and fed DoE studies.

**Table 1:** Composition and concentration levels employed in full range design of experiment.

Parameter	Substance	Lower limit fasted	Upper limit fed
Bile salt (mM)	Sodium taurocholate	1.5	24
Lecithin (mM)	Phosphatidylcholine	0.2	4.8
Fatty acid (mM)	Sodium oleate	0.5	52
pH	Sodium hydroxide/hydrochloric acid	5	7
Salt (mM)	Sodium chloride	68	203
Buffer (mM)	Phosphate^a^	15	45
Monoglyceride (mM)	Glyceryl mono-oleate	0.5	6.5

a Monophosphate buffer (KH_2_PO_4_).

## 2 Materials and methods

### 2.1 Materials

Sodium taurocholate, ammonium formate, sodium chloride (NaCl), chloroform, formic acid, monosodium phosphate (NaH_2_PO_4_), fenofibrate, indomethacin and phenythoin were purchased from Sigma Aldrich Poole, Dorset UK. Lecithin S PC (phosphatidylcholine from Soybean “98%”) was purchased from Lipoid. Glycerol mono oleate was obtained from CRODA Healthcare. The active pharmaceutical ingredients felodipine, probucol, aprepitant, tadalafil, carvedilol and zafirlukast were provided through OrBiTo by Dr. R. Holm Head of Preformulation, Lundbeck, Denmark. Sodium oleate was obtained from BDH Chemical Ltd. Poole England. The analytical solvents methanol and acetonitrile were of HPLC grade (VWR, UK). All water was ultra pure Milli-Q water.

### 2.2 Design of experiment and data analysis

A quarter of the full factorial design of experiment with 7 factors (either a component concentration or a system parameter such as pH) and 2 levels (upper and lower limits) was constructed and analysed using Minitab®17.2.1. Minitab generated 32 different experiments by various combinations of the upper and lower limits of the 7 factors based on Table 1 (no centre point and no replicate). When designing and analysing the DoE assumptions were made. 1. Only main effects and 2-way interactions are considered in the analysis and 3-way interactions or more were not considered. 2. The single factors and factor interactions are confounded with 3 to 6-way interactions which were not included. There are three confounded 2-way factor interactions, sodium oleate and salt with buffer and monoglyceride, sodium oleate and buffer with salt and monoglyceride, sodium oleate and monoglyceride with salt and buffer. For these interactions if the result is significant then any conclusions must be drawn with caution as it might be the result of the four factors together or only one of the 2 way interactions. 3. The main effect can be positive (+) or negative (−), but when it is involved in an interaction, the conclusion will be considered with the interactions (±).

The Kolmogorov normality test was used in Minitab® to assess the distribution of each data set, based on the result that all data sets have a non-normal distribution the Mann-Whitney test was applied to evaluate differences between two data sets.

### 2.3 Equilibrium solubility measurements

#### 2.3.1 Preparation of lipid stock mixtures

Sodium taurocholate, monoglyceride and lecithin were weighed into a flask and 2 ml of chloroform was added to dissolve all the solid material. A stream of nitrogen gas was used to remove the chloroform ensuring a dry film was produced. Water was added to reform the dried film, stirred to obtain a homogenous mixture, transferred to a volumetric flask (5 ml) and made to volume with water.

#### 2.3.2 Preparation of aqueous stock solutions

Salt and buffer stock solution: Sodium chloride (4.448 g) and monosodium phosphate (NaH_2_PO_4_) (2.395 g) were weighed into a 25 ml volumetric flask, dissolved and made up to the volume with water.

Sodium oleate: Sodium oleate (1.978 g) was weighed into a 25 ml volumetric flask, dissolved in water under gentle heat and made to final volume. Solution was then kept at 50 °C to aid solubilisation.

#### 2.3.3 Preparation of measurements solutions

The concentration of each stock mixture has been designed to be 15 times greater than the upper limit concentration value required for the DoE, with the exception of sodium oleate where only a 5 times concentration was possible. The stock mixtures were combined to provide the 32 measurement solutions according to the DoE model.

#### 2.3.4 Determination of equilibrium solubility

This protocol has been previously validated to ensure equilibrium solubility is achieved after 24 h with no methodological interference (Khadra et al., 2015, Zhou et al., 2017). A weight of powdered drug (10 mg) was added to a centrifuge tube (15 ml Corning®). The required volume of each stock solution (section above) and water was added to provide a final volume of 4 ml in every tube. pH was then adjusted to 5 or 7 using 0.1 M HCL or 0.1 M KOH. Tubes were shaken for 1 h at room temperature and then pH adjusted again as before. Tubes are then placed in an orbital shaker and incubated for 24 h at 37 °C and 240 rpm. Following incubation the tubes were checked for the presence of solid drug, then centrifuged (13,000 rpm, 5 min) and the supernatant (500 μl) was sampled to determine the solubilised drug concentration by HPLC. Assays conditions are presented in Table 2.

**Table 2:** HPLC assays conditions.

Column^a^	Drug	Mobile phase	Flow rate (ml/min)	Injection volume (μl)	Detection (nm)	Retention time (min)	R^2^^b^	LOQ (μM)
2	Indomethacin	Mobile phase A: Ammonium formate 10 mM pH 3.0 in H_2_O Mobile phase B: Ammonium formate 10 mM pH 3.0 in ACN/H_2_O (9:1 v/v)	1	10	254	0.84	0.9919	0.31
2	Phenytoin		1	10	260	2.3	0.9961	51
1	Felodipine		1	10–50	260	3.1	0.9997	9.4
1	Fenofibrate		1	10	291	3.7	0.994	0.94
1	Probucol		1	10–50	254	4.5	0.9999	2.5
1	Aprepitant		1	10	254	3.0	0.9995	26
2	Tadalfil		1	10	291	2.1	0.998	1.9
2	Zafirlukast		1	10	260	2.9	0.9991	0.27
2	Carvedilol		1	10	254	1.0	0.9999	9.0

Apparatus Agilent Technologies 1260 Series Liquid Chromatography system with Clarity Chromatography software: Gradient method: Time 0, 70%A:30%B, 3 min 0%A:100%B, 4 min 0%A:100%B, 4.5 min 70%A:30%B total run time 8 min. ACN: acetonitrile. LOQ: Limit of Quantification.

a Column 1 Hichrom ACE 3 C18/DV148262/50 × 3.0 mm id/ACE-111-0503/A149937: Column 2 Hichrom ACE 3 C18/SIN-A46224/50 × 2.1 mm/ACE-111-0502/A46224.

b *R*^2^ Linear regression coefficient of calibration curve, *n* = 5 or 6 points.

## 3 Results and discussion

### 3.1 Equilibrium solubility measurements

**Fig. 1:**
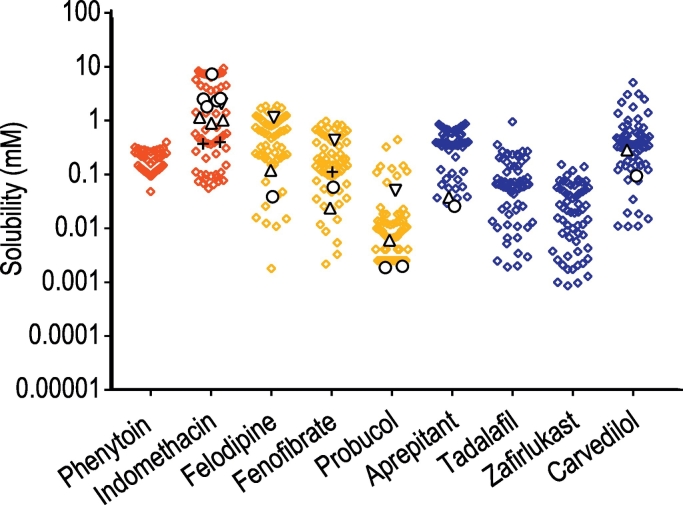
Design of experiment equilibrium solubility measurements. Legend: Equilibrium solubility measurements for each drug in DoE media compositions detailed in Table 1. Red coloured data points for acidic drugs, yellow for neutral drugs and blue basic drugs. **∆** and **+** reported solubility values for individual drugs in fasted simulated intestinal fluid and fed simulated intestinal fluid media respectively, **○** and **▽** reported solubility values for individual drugs in fasted human intestinal fluid and fed human intestinal fluid respectively, all values from (Augustijns et al., 2014). (For interpretation of the references to colour in this figure legend, the reader is referred to the web version of this article.)

The results of the full range DoE equilibrium solubility measurement are presented in Fig. 1 and a broad range of solubility values are observed with heterogeneous variability from one to three orders of magnitude depending on the drug. As a comparison literature solubility values were available for six drugs in fasted or fed state simulated intestinal fluid (SIF) and/or human intestinal fluid (HIF) (Augustijns et al., 2014) and are plotted in Fig. 1. Those results are comparable in each case and lie within the DoE range of the solubility values reported in this study. It is evident that drug specific factors are affecting solubility with some compounds felodipine and tadalafil showing a large variability while phenytoin and aprepitant show more consistency.

**Fig. 2:**
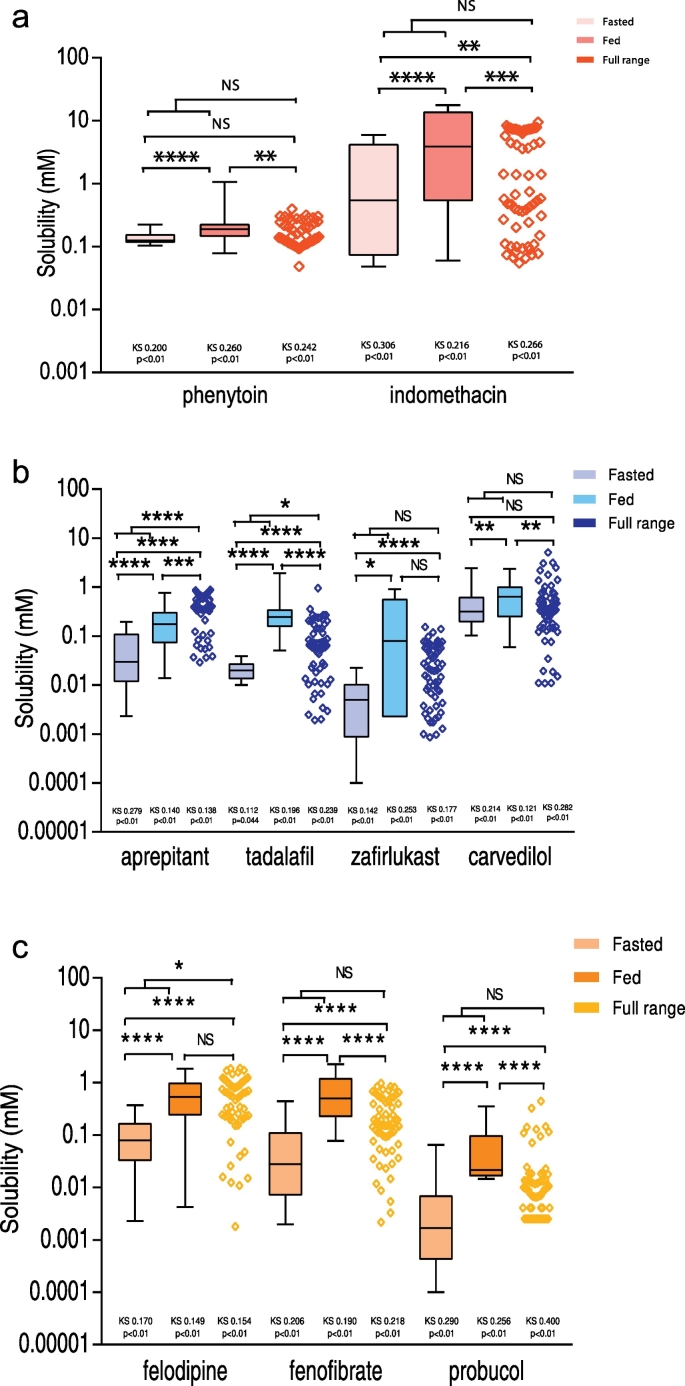
Statistical comparison of design of experiment equilibrium solubility measurements. a: Acidic drugs. Legend:  Equilibrium solubility measurements for each drug in full range design of experiment media. Box and whisker plots from top to bottom the maximum value, 75th percentile, median, 25th percentile and minimum value,  fasted design of experiment solubility data (Khadra et al., 2015);  fed design of experiment solubility data (Zhou et al., 2017). KS statistic value of Kolmogorov normality test on the data set, if *p*-value is < 0.05 the distribution is non-normal. Mann-Whitney non parametric test determine whether the population medians of two groups differ, not significant (NS) if *p*-value > 0.05; * if *p*-value ≤ 0.05; ** if *p*-value ≤ 0.01; *** if *p*-value ≤ 0.001 and **** if *p*-value ≤ 0.0001. b: Basic drugs. Legend:  Equilibrium solubility measurements for each drug in full range design of experiment media. Box and whisker plots from top to bottom the maximum value, 75th percentile, median, 25th percentile and minimum value,  fasted design of experiment solubility data (Khadra et al., 2015);  fed design of experiment solubility data (Zhou et al., 2017). KS statistic value of Kolmogorov normality test on the data set, if *p*-value is < 0.05 the distribution is non-normal. Mann-Whitney non parametric test determine whether the population medians of two groups differ, not significant (NS) if *p*-value > 0.05; * if *p*-value ≤ 0.05; ** if *p*-value ≤ 0.01; *** if *p*-value ≤ 0.001 and **** if *p*-value ≤ 0.0001. c: Neutral drugs. Legend:  Equilibrium solubility measurements for each drug in full range design of experiment media. Box and whisker plots from top to bottom the maximum value, 75th percentile, median, 25th percentile and minimum value,  fasted design of experiment solubility data (Khadra et al., 2015);  fed design of experiment solubility data (Zhou et al., 2017). KS statistic value of Kolmogorov normality test on the data set, if *p*-value is < 0.05 the distribution is non-normal. Mann-Whitney non parametric test determine whether the population medians of two groups differ, not significant (NS) if *p*-value > 0.05; * if *p*-value ≤ 0.05; ** if *p*-value ≤ 0.01; *** if *p*-value ≤ 0.001 and **** if *p*-value ≤ 0.0001.

In Fig. 2a–c the published fasted (Khadra et al., 2015) and fed (Zhou et al., 2017) DoE measurements are plotted for each drug along with the full range data and displayed by group. For acidic drugs (Fig. 2a) the concentration points are comparable with the previous fasted and fed studies with the solubility of phenytoin very consistent while indomethacin exhibits a larger variability. The respective pKa of the drugs 8.1 and 4.5 could explain this difference since phenytoin is un-ionised over the experimental pH range whilst indomethacin is predominantly ionised. In addition Indomethacin is more lipophilic (log *P* = 4.3) which will increase its interaction with the micellar phase. For the basic drugs (Fig. 2b) tadalafil and carvedilol the previous fasted and fed data are comparable to the full range experiment whilst zafirlukast and aprepitant do not show the same consistency. The full range DoE was not able to determine the lowest concentrations for both drugs. Zafirlukast has the biggest difference between the lowest fasted and highest fed values (4 orders of magnitude). In addition the distribution of the full range data points is the most homogenous compared to the other distributions. This compound has the highest log *P* value (5.4) and a pKa of 4.3 which means that the non-ionised form is predominant over the pH range. Carvedilol and tadalafil are largely ionised between pH 5 and 7 according to their respective pKa values of 7.8 and 10 while the ampholyte Aprepitant is considered as a neutral compound between pH 5 and 7. The lipohilicity of aprepitant (log *P* = 4.5) and carvedilol (log *P* = 4.2) could explain the slightly higher solubility observed. For neutrals (Fig. 2c) depending on the drug the full range experiment was able to determine very low concentrations. Neutral compounds are not ionisable therefore lipophilicity plays an important role in the solubilisation by surfactants and micelles. Felodipine and fenofibrate (log *P* = 3.8 and 5.2 respectively) behave similarly since the full range covered the fasted and fed space and the lowest concentration corresponds to the lowest point of the fasted experiment. However, the solubility of probucol is lower which may indicate that its very high lipophilicity (log *P* = 10.9) might limit solubilisation. Interestingly the measured equilibrium solubility values indicate that the full range DoE covered the solubility space of the previous fasted and fed DoE for the majority of the drugs. This outcome means the full range DoE is covering an appropriate solubility space and that a reduced experimental size DoE could be sufficient to explore the intestinal solubility variability in simulated media.

### 3.2 Statistical comparisons

All the data sets resulted in a non-normal distribution, which based on the number of data points (fasted DoE = 66 (Khadra et al., 2015), fed DoE = 92 (Zhou et al., 2017), full range = 32) was not expected and may arise either through the non-normal sample pattern induced by the DoE structure and or the fact that drug solubility is not normally distributed in the sample space. The latter explanation is supported by human intestinal fluid characterization studies which indicate that bile salt and lecithin in the fasted state have skewed concentration distributions (Riethorst et al., 2015) and HIF solubility studies measuring differences between mean and median solubility values (Psachoulias et al., 2011) indicating a non-normal solubility distribution. Further studies will be required to fully explore this interesting statistical property. A non-parametric Mann-Whitney test was therefore applied to compare distributions and the *p*-values are displayed in Fig. 2a–c.

It is evident for all the drugs that the solubility values are statistically significantly lower in the fasted than the fed state which is in agreement with the literature data (Augustijns et al., 2014, Bevernage et al., 2010, Clarysse et al., 2011) and indicates that the published DoE (Khadra et al., 2015, Zhou et al., 2017) studies have explored different solubility spaces. A comparison of the published fasted or fed solubility distributions with the current full range DoE indicates that there is a statistically significant difference for fourteen (approximately 80%) out of the possible eighteen (two for each of the nine drugs tested) comparisons. In the fasted state phenytoin and carvedilol and in the fed state zafirlukast and felodipine are statistically equivalent to the combined DoE. The difference between fasted or fed compared to combined is to be expected based on the previous comparison between fasted and fed, which determined that these are separate solubility distributions. In the case of phenytoin and carvedilol in the fasted and felodipine in the fed DoE the similarity can be ascribed to the narrow solubility distribution, which fits inside the full range distribution, whilst for zafirlukast there is a broad overlapping between fed and full range.

A comparison of the combined fasted and fed solubility data, which if additive should represent the full solubility range, with the full range DoE indicates that there is no significant difference for six (phenytoin, indomethacin, zafirlukast, carvedilol, fenofibrate, probucol) out of the nine drugs tested but aprepitant, tadalafil and felodipine are significantly different. The statistical equivalence between a combination of published fasted and fed data with the full range DoE data is to be expected if both experiments are sampling the same solubility space. The statistically significant difference determined for aprepitant, tadalafil and felodipine appears to be related to the trend towards higher “fed” like solubility values in the full range DoE, when compared to the solubility for the combined fasted and fed data. However, for aprepitant and tadalafil there is a statistically significant difference between all data sets (Fig. 2b) indicating that these drugs are exhibiting complex behaviour. This discrepancy in one third of the tested drugs might be due to the aforementioned issue that the application of a DoE approach samples the solubility space in a structured rather than random fashion and therefore statistical comparison might not be valid. Conversely, two thirds of the tested drugs behave in a manner that is consistent with published paradigms.

The current solubility results match literature data (Fig. 1) where available indicating that the DoE approaches are investigating a relevant solubility zone, but there are no equivalent large literature data sets available for statistical comparison. Almost all published solubility studies in either human intestinal fluids (Augustijns et al., 2014, Clarysse et al., 2009, Kleberg et al., 2010) or simulated intestinal fluids (Clarysse et al., 2011, Fuchs and Dressman, 2014, Ilardia-Arana et al., 2006, Khadra et al., 2015, Zhou et al., 2017) indicate that there are drug dependent variations in solubility over and above those induced by variations in media composition. In combination with the results in this study this indicates that a substantial proportion, around one third, of drugs most probably basic or neutral compounds, will exhibit behaviour at the extremes of current literature based patterns.

### 3.3 Solubility influence of individual DoE factors

**Fig. 3:**
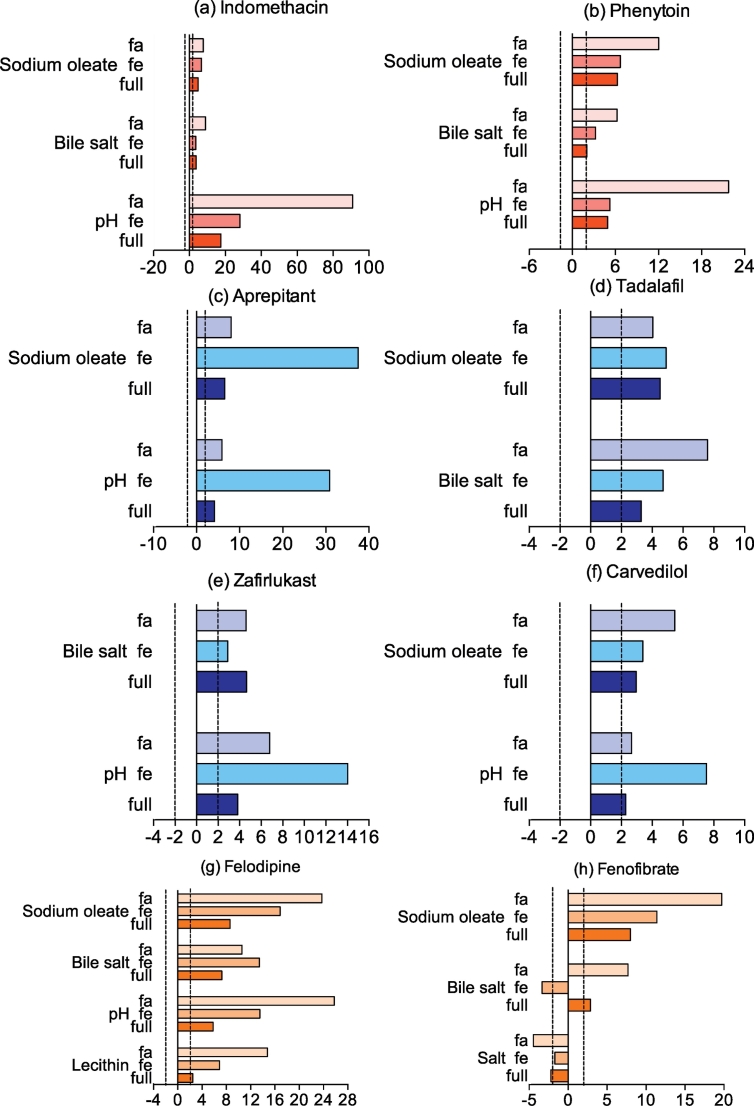
Statistically significant standardised effect values for individual DoE factors on equilibrium solubility. Legend: DoE standardised effect values for individual factors (as listed in figure y-axis) on equilibrium solubility. Vertical black lines indicate statistical significance (*p* < 0.05), horizontal bar direction indicates direction of effect, to the right of 0 on axis is positive effect on solubility, bar length indicates the magnitude of the effect. Full: value from current study, Fa: fasted data from (Khadra et al., 2015), Fe: fed data from (Zhou et al., 2017). NB For each drug non-statistically significant factor effects in this study are not presented.

For each DoE experiment Minitab calculates an individual factor's standardised effect on the magnitude and direction of the measured equilibrium solubility, allowing a comparison between factors and drugs. For each drug, statistically significant standardised effect values in the full range study are presented in Fig. 3 along with the standardised effect value for that factor in the published fasted and fed studies (Khadra et al., 2015, Zhou et al., 2017). NB For each drug non-statistically significant factor effects in this study are not presented in Fig. 3, which does not mean that a statistically significant effect was not determined in either the fasted or fed study.

**Fig. 4:**
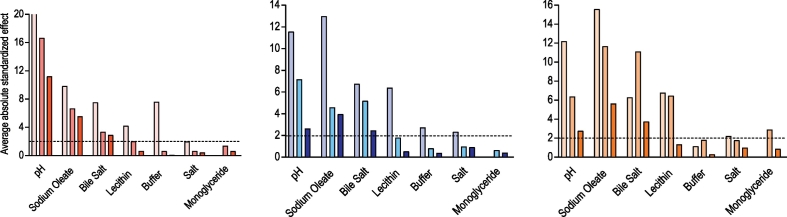
Average absolute standardised effect values for individual DoE factors on equilibrium solubility. Legend: Average absolute (NB this removes direction of effect information) standardised effect values for individual factors on equilibrium solubility grouped by drug category. Horizontal black line indicates statistical significance (*p* < 0.05). Acidic Drugs;  current full range study,  fasted design of experiment solubility data (Khadra et al., 2015);  fed design of experiment solubility data (Zhou et al., 2017). Basic Drugs;  current full range study,  fasted design of experiment solubility data (Khadra et al., 2015);  fed design of experiment solubility data (Zhou et al., 2017). Neutral Drugs;  current full range study,  fasted design of experiment solubility data (Khadra et al., 2015);  fed design of experiment solubility data (Zhou et al., 2017).

The effects of the full range media factors on the drugs are complex because each drug displays a unique profile a result that is similar to the previous fasted (Khadra et al., 2015) and fed (Zhou et al., 2017) studies. The media components showing the lowest effect on the solubility are buffer and monoglyceride (0 significant results from 9 drugs) followed by salt and lecithin (1 significant from 9), whilst the factors with the biggest influence are pH, bile salt (6 significant results from 9) and sodium oleate (7 from 9). This is comparable to the fasted and fed state DoE where bile salt, pH and sodium oleate were the dominant significant factors but contrasting for lecithin which was also significant in these studies. However, the amplitude of the effect differs between groups and individual drugs confirming the complexity of the interplay between each drug and the system, a feature higlighted in both previous DoE studies. The means of the absolute standardised effect values grouped for acidic, basic and neutral drugs are presented in Fig. 4. This provides information on the overall magnitude of a factors influence but masks the direction of the effect. For the three groups of drugs pH, sodium oleate and bile salt have a statistically significant influence on solubility in the full range study which is in accordance with the previous reported experiments (Khadra et al., 2015, Zhou et al., 2017).

For acidic compounds (Fig. 3a–b) pH is the most significant factor, which is identical to the two previously reported DoE studies and has already been described for acidic compounds (Clarysse et al., 2009). The direction of effect is comparable (positive) but the magnitude is lower when compared to the published fasted study and similar to the fed study. Sodium oleate and bile salt are the second most significant factors with a positive direction of effect, which is in agreement with the published fasted and the fed state. On the contrary buffer had no influence even though it was significant for the two compounds in the fasted study with a positive effect for phenytoin and a negative effect for indomethacin. The influence of the remaining factors (lecithin, salt and monoglyceride) is also negative.

For basic compounds (Fig. 3c–f) sodium oleate, pH and bile salt are the predominant factors but this effect is variable between drugs. Aprepitant, carvedilol and tadalafil are positively affected by sodium oleate whilst bile salt only positively affects tadalafil and zafirlukast. The influence of pH is not as important as for acidic drugs with a significant effect featured for aprepitant, carvedilol and zafirlukast but not tadalafil. An enhanced solubility is coherent with an increase of pH from 5 to 7 for the weak base zafirlukast, pKa value of 4.3 influencing drug ionisation but for aprepitant (pKa = 9.7) this solubility change has to arise via another mechanism. Surprisingly lecithin was not significant which is at variance from the published DoE where sodium oleate, bile salt, pH and lecithin were significant for basic drugs.

For neutral compounds (Fig. 3g and h) only felodipine and fenofibrate were significantly affected by any factors. Sodium oleate and bile salt had a positive effect on both drugs whilst pH and lecithin only affected felodipine in both cases in agreement with both studies in fasted and fed states. On the other hand probucol was not significantly influenced by any of the factors although previously oleate, bile salt, pH, lecithin salt and monoglyceride were detected as significant. The influence of pH cannot change drug ionisation therefore for these drugs the solubility influence has to be associated with a change in ionisation of the media components as presented previously (Khadra et al., 2015, Pedersen et al., 2000a, Zhou et al., 2017). Finally buffer, salt and monoglycerides showed a very small influence on solubility with very low magnitude mostly below the significant level, reflecting the fasted study but contrasting with the fed study where almost all the components were significant.

### 3.4 Solubility influence of DoE factor interactions

**Fig. 5:**
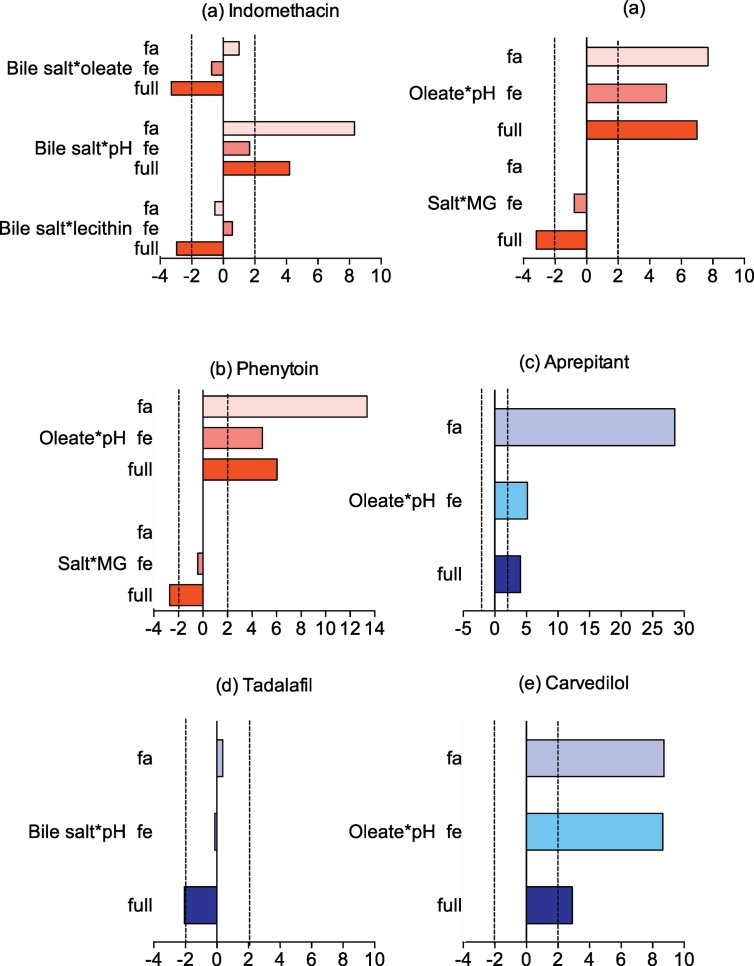

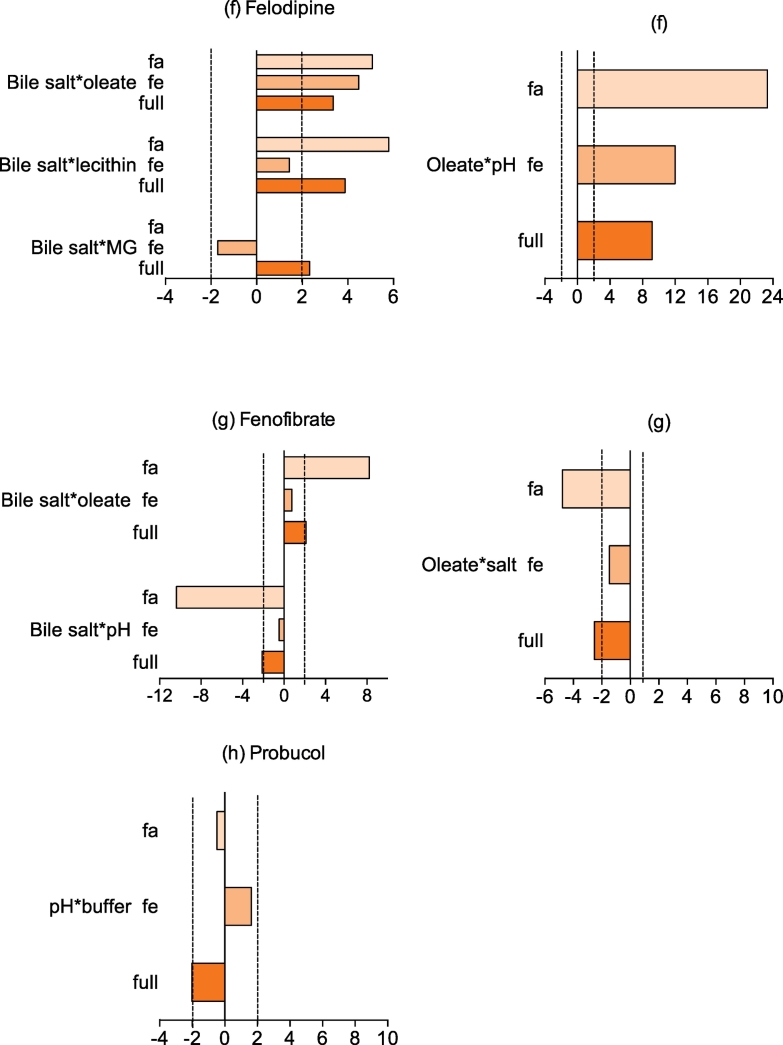
Standardised effect values for DoE factor interactions on equilibrium solubility. Legend: DoE standardised effect values (x-axis) for interactions between factors (as listed in figure titles) on equilibrium solubility in the fasted (fa), fed (fe) and full range (full) experiment. Vertical dashed black lines indicate statistical significance (*p* < 0.05), bar direction indicates direction of effect, to the right of 0 on x axis is positive effect on solubility, bar length indicates the magnitude of the effect. Full: value from current study, Fa: fasted data from (Khadra et al., 2015), Fe: fed data from (Zhou et al., 2017). NB For each drug non-statistically significant factor interactions in this study are not presented.

The experiment consisted of seven factors and a possible twenty one interactions between the factors. Only 2-way interactions were considered. Three confounded interactions are present, sodium oleate and salt with buffer and monoglyceride, sodium oleate and buffer with salt and monoglyceride, sodium oleate and monoglyceride with salt and buffer. For each drug, statistically significant standardised effect values for factor interactions in the full range study are presented in Fig. 5 along with the standardised effect value for that factor interaction in the published fasted and fed studies (Khadra et al., 2015, Zhou et al., 2017). NB For each drug non-statistically significant factor interactions in this study are not presented in Fig. 5, which does not mean that a statistically significant effect was not determined in either the fasted or fed study. Among all the possible factor combinations in this full range DoE a statistically significant effect was present eighteen times which represents approximately 10% of the possibilities. For neutral and acidic drugs eight and seven significant interactions featured respectively while for basic drugs only three. This contrasts with the fasted and fed DoE where respectively one third and one fifth of the interactions were significant.

For acidic drugs (Fig. 5a–b) the effect of factor interactions is consistent within the group, bile salt or sodium oleate are associated in each significant interaction and pH is only present in three of them. The limited effect of pH is surprising since the pKa values of the two acidic factors (oleate and bile salt) is approximately 5 (Holm et al., 2013) and the DoE range is 5–7 which must induce variation in factor ionisation. Interestingly the combination of salt and monoglyceride is significant for both compounds, which could be a confounded effect since this interaction is linked with sodium oleate and buffer. For basic compounds (Fig. 5c–e) only three significant interactions were highlighted and they all include pH with either bile salt or sodium oleate. This is expected as these factors were predominant in the single factor analysis and also reported during the fasted and fed DoE. For neutrals drugs (Fig. 5f–h) all the significant interactions are associated with bile salt or sodium oleate. Although the oleate and salt interaction for fenofibrate is confounded with buffer and monoglyceride. The positive effect of surfactant has been previously reported in the fasted and fed DoE for this group of drugs.

### 3.5 Statistically significant solubility factor and factor interactions

**Fig. 6:**
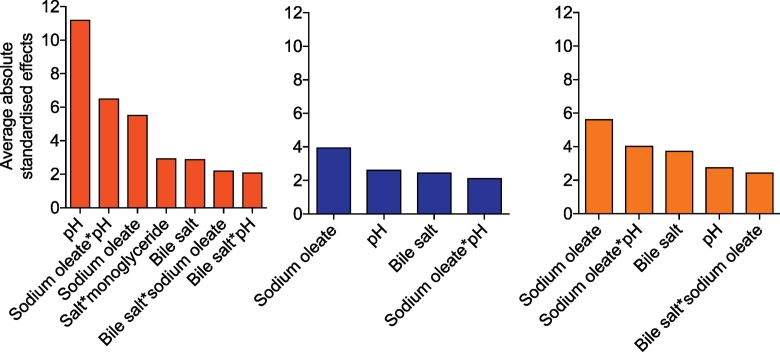
Average significant absolute standardised effect values for individual factors and factor interactions. Legend: Average significant DoE standardised effect values for individual factors and factor interactions (as listed in figure x-axis) on equilibrium solubility grouped by drug category. Red coloured bars for acids, blue for basics and yellow for neutrals. NB Only statistically significant results presented. (For interpretation of the references to colour in this figure legend, the reader is referred to the web version of this article.)

The mean of the absolute effect value of all statistically significant factor and factor interactions arranged by drug group is presented in Fig. 6 in order to summarise the full range experimental results. When comparing factor interactions between this full range and published results conclusions should be made with prudence since the fed study employed a different statistical design of experiment (Zhou et al., 2017). For the acidic drugs pH is not surprisingly the principal factor. In the published fasted (Khadra et al., 2015) and fed (Zhou et al., 2017) study pH is involved in every statistically significant combination with either sodium oleate, bile salt or buffer. These interactions are confirmed in the full range experiment as pH, sodium oleate and bile salt are responsible for three out of four. The significant interaction of salt with monolgyceride is a result that is not present in the published fed DoE (Zhou et al., 2017) but is a confounded interaction in this study with sodium oleate with buffer, it is therefore likely that this is due to a dominant effect arising from sodium oleate. For the basic drugs sodium oleate, pH, bile salt and the interaction between sodium oleate and pH was found to be statistically significant. Interestingly those components are involved in the two significant interactions highlighted in the fed DoE (pH ∗ sodium oleate and lecithin ∗ sodium oleate) (Zhou et al., 2017). However, in the fasted DoE six different interactions were significant, pH with sodium oleate, salt and lecithin, bile salt with sodium oleate and buffer and then lecithin with salt. For the neutral drugs the previous fasted and fed studies had described a more complex pattern with eight and fifteen significant interactions respectively. Surprisingly the full range experiment is not reflecting this result since only two significant factors were present (sodium oleate with pH and bile salt). The reduced experiment full range DoE is therefore picking up fewer significant factors and factor interactions than the larger focussed experimental studies.

### 3.6 Comparison of full range DoE with published fasted and fed DoE

For each compound the significance of individual media factors standardised effect on the equilibrium solubility is presented in Table 3 juxtaposed to the published fasted and fed DoE results. The factor least consistent with previous studies is lecithin (2 matches from 9 drugs) followed by buffer (3 matches from 9 drugs), salt, monoglyceride and bile salt are intermediate (4 or 5 matches from 9 drugs) with pH (6 matches from 9 drugs) and oleate (7 matches from 9 drugs) the most consistent. In addition for any factor the full range study has the lowest number of significant findings when compared to the published studies a result also applicable to two way factor interactions. The difference in the ability to detect the significance of a factor's contribution to equilibrium solubility may be due to a number of differences between the studies. The reduced number of sample points for the full range (32 vs 66 (fasted) or 92 (fed)) study must reduce the statistical resolution and therefore only factors which are highly significant or not significant are detected correctly, see Fig. 4 and Table 3. In addition a design of experiment statistically combines high and low levels of a factor to construct the measurement points, covering the full range (fasted to fed) will produce factor ratios that are not likely to be biorelevant (Riethorst et al., 2015). This may be why lecithin has the lowest consistency since the influence of lecithin observed in the previous published DoEs (Khadra et al., 2015, Zhou et al., 2017) was the least significant it is not reproduced or captured in this full range study. The importance of the “solubilizing” capacity (combination of bile salt, lecithin, and sodium oleate) has been reported to significantly enhance solubility (Kleberg et al., 2010, Pedersen et al., 2000b, Soderlind et al., 2010) but this is not evident in this study. Further more detailed studies with increased drug numbers and properties along with scaled experimental number design of experiment approaches would be required to fully elucidate the reasons for these findings.

**Table 3:**
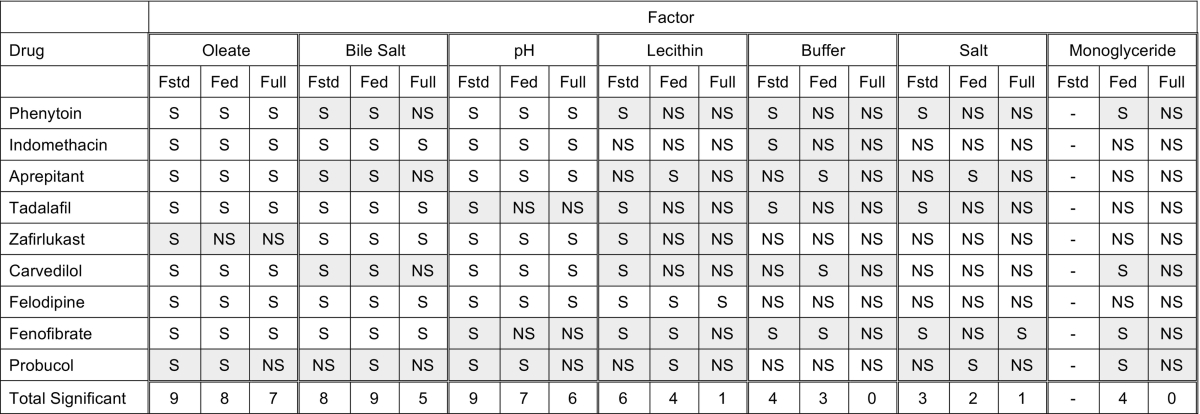
Comparison of the statistical significance of DoE factors.

Fstd = fasted media design of experiment (Khadra et al., 2015 #9648).

Fed = fed media design of experiment (Zhou et al., 2017 #10289).

Full = full range media design of experiment.

- monoglyceride not included in fasted media design of experiment.

S = factor statistically significant in design of experiment study.

NS = factor not statistically significant in design of experiment study.

Shaded box = no consistent result between studies.

## 4 Conclusions

The objective of this study was to determine the feasibility of combining the previous fasted and fed DoE into one smaller full range experiment in order to obtain comparable results regarding the influence of gastrointestinal media components on the equilibrium solubility of BCS class II drugs. This full range DoE produced interesting results regarding the general solubility space, which overall is comparable to published fasted (Khadra et al., 2015), fed (Zhou et al., 2017), simulated and human intestinal fluid equilibrium solubility values (Augustijns et al., 2014). A statistical comparison of the published fasted and fed solubility distributions indicates that these are significantly different, with the fed higher than the fasted a result which is in agreement with literature data based on single measurements (Augustijns et al., 2014). A statistical comparison of the full range measured equilibrium solubility values with a combination of the published fasted and fed indicates that this full range experiment was statistically equivalent to the previous fasted and fed DoE for six out of the nine drugs tested (Fig. 2). However, for three drugs statistically significant differences were detected indicating that for these drugs the full range DoE will not provide equivalent equilibrium solubility information to separate larger studies. It is likely that this behaviour will be present in other drugs which will be most probably be either basic or neutral in character, however further research is required to fully elucidate the molecular properties that produce this effect. The measured solubility distributions for each drug in the full range experiment and the published fasted and fed experiments was non-normal a result that may be due to the structured sampling induced by the DoE, the fact that the distribution is non-normal either through the presence of multiple distributions or extreme points or that the number of data points is not sufficient to sample the distribution. Further studies will be required to determine the origin of this result however it implies that a single solubility measurement without knowledge of the solubility distribution will be of limited value.

Overall the three drug groups exhibited a similar profile with respect to the most significant factors and two way factor interactions controlling solubility when compared to the published fasted and fed studies. For acidic compounds unsurprisingly pH and oleate were dominant (Khadra et al., 2015, Zhou et al., 2017) with bile salt also significant. For the neutral and basic drugs three factors pH, bile salt and sodium oleate were dominant along with a two way interaction of sodium oleate with pH and for neutral drugs only bile salt with sodium oleate. Although there was variation between the drugs the four other factors (lecithin, monoglyceride, salt and buffer) were on average not significant at all along with around 90% of possible two way factor interactions. The reduced incidence of significant effects with individual factors and two way factor interactions may be a consequence of the reduced number of measurement points within the design of experiment and or the combination of factor values covering the fasted and fed range leading to systems that are not biorelevant. Notably, lecithin did not significantly influence solubility a result that contrasts with previous published information as it is reported to be essential in the solubilizing capacity of simulated intestinal fluids (Soderlind et al., 2010) and was significant in published DoE studies (Khadra et al., 2015, Zhou et al., 2017).

The reduced experiment full range DoE study therefore provides equilibrium solubility values that for the majority of drugs will be equivalent to larger studies but with a lower statistical ability to identify the significant factors and factor interactions that influence solubility. Further statistical refinement might be possible to tease out the differences between the fasted and fed states using for example a dual small scale DoE covering both states. This might also provide information to determine why some drugs do not produce equivalent solubility results between DoE approaches.

## Footnotes

1 Aprepitant has been classified as a basic drug in order to assist comparison with the two previous Design of Experiment studies (Khadra et al., 2015, Zhou et al., 2017), it is recognised that with a reported pKa of 9.7 (Liu et al., 2015) at the pH values in this study it will be predominantly un-ionised.
